# Getting the Balance Right: A randomised controlled trial of physiotherapy and Exercise Interventions for ambulatory people with multiple sclerosis

**DOI:** 10.1186/1471-2377-9-34

**Published:** 2009-07-16

**Authors:** Susan Coote, Maria Garrett, Neasa Hogan, Aidan Larkin, Jean Saunders

**Affiliations:** 1 Department of Physiotherapy, University of Limerick, Limerick, Ireland; 2 Project Coordinator, Multiple Sclerosis Society of Ireland, Galway, Ireland; 3 Statistical Consulting Unit, University of Limerick, Limerick, Ireland

## Abstract

**Background:**

People with Multiple Sclerosis have a life long need for physiotherapy and exercise interventions due to the progressive nature of the disease and their greater risk of the complications of inactivity. The Multiple Sclerosis Society of Ireland run physiotherapy, yoga and exercise classes for their members, however there is little evidence to suggest which form of physical activity optimises outcome for people with the many and varied impairments associated with MS.

**Methods and design:**

This is a multi-centre, single blind, block randomised, controlled trial. Participants will be recruited via the ten regional offices of MS Ireland. Telephone screening will establish eligibility and stratification according to the mobility section of the Guys Neurological Disability Scale. Once a block of people of the same strand in the same geographical region have given consent, participants will be randomised. Strand A will concern individuals with MS who walk independently or use one stick to walk outside. Participants will be randomised to yoga, physiotherapy led exercise class, fitness instructor led exercise class or to a control group who don't change their exercise habits.

Strand B will concern individuals with MS who walk with bilateral support or a rollator, they may use a wheelchair for longer distance outdoors. Participants will be randomised to 1:1 Physiotherapist led intervention, group intervention led by Physiotherapist, group yoga intervention or a control group who don't change their exercise habits. Participants will be assessed by physiotherapist who is blind to the group allocation at week 1, week 12 (following 10 weeks intervention or control), and at 12 week follow up. The primary outcome measure for both strands is the Multiple Sclerosis Impact Scale. Secondary outcomes are Modified Fatigue Impact Scale, 6 Minute Walk test, and muscle strength measured with hand held dynamometry. Strand B will also use Berg Balance Test and the Modified Ashworth Scale. Confounding variables such as sensation, coordination, proprioception, range of motion and other impairments will be recorded at initial assessment.

**Discussion:**

Data analysis will analyse change in each group, and the differences between groups. Sub group analysis may be performed if sufficient numbers are recruited.

**Trial registration:**

ISRCTN77610415

## Background

Multiple sclerosis (MS) is a demyelinating, degenerative disease of the central nervous system. It can cause a multitude of motor, sensory, visual, psychological, sexual, and bladder and bowel symptoms. Europe has the highest estimated prevalence of MS in the world at 80 per 100,000[1] and in Ireland people with MS (PwMS) make up the largest diagnostic group (9.2%) of those registered on the National Physical and Sensory Disability Database[2]. MS is a progressive disease that can lead to disability, functional limitations and a poor quality of life (QoL). In a recent profiling study[3], 59% of the people with MS had EDSS scores between 0 and 4.0, 18% had scores of 6.0 ± 6.5, and 6% had a score of 8.0. It is suggested that within 15 – 25 years of diagnosis nearly 50% of PwMS will reach an EDSS score of at least 6 meaning they will require the use of a walking aid[4,5]. PwMS have a normal life expectancy, therefore, they may have to live for many years with severe mobility problems and have a need for regular therapeutic intervention. Therefore the importance of appropriate and timely intervention in patients with relapsing disease to slow or prevent the accumulation of physical disability associated with progressive types of disease is apparent.

There is a significant body of evidence to suggest that exercise programmes have a beneficial effect on both disease symptoms and general fitness of people with MS who are ambulatory [6]. The authors recommend that future studies should adhere to methodological principles of allocation concealment, blind recording and description of dropouts.

A meta-analysis of physical activity levels of people with MS concluded that they engage in significantly less physical activity than non-diseased populations[7]. In a study investigating coronary heart disease risk in women with MS, those who had higher levels of leisure time physical activity had a lower risk of secondary disease[8] and ambulatory women with MS who took part in a resistance training programme also had a decreased risk of coronary artery disease[9]. A meta analysis of the relationship between exercise and quality of life for people with MS suggested that quality of life can be optimally improved through exercise programmes less than three months duration, involve greater than 90 minutes a week, and be evaluated using MS specific measures[10]. Given the positive relationship between physical activity and improving disease symptoms and quality of life, and reducing secondary disease, it is essential that the optimal physical activity programmes are identified.

In physiotherapy practice it is acknowledged that people with differing levels of mobility will have varying treatment needs therefore the literature has been separately reviewed for those with an EDSS of 0–6 and for those with an EDSS of >6.

The literature published since the Cochrane review[6] on the specific benefits of physiotherapy and exercise interventions for PwMS with an EDSS of 0–6 suggests that aerobic exercise positively influences fitness, walking speed, gait parameters, disability, fatigue and quality of life [11-16]. Additionally, Progressive Resistance Exercise (PRE) improved strength, walking over short distances, improved stepping time, improved fatigue and reduced the physical impact of MS[16]. However, there was only one intervention followed up at three months [17-20] and this found that benefits were maintained for the Functional Assessment of MS and the MSIS-29 only.

Several studies have combined aerobic exercise and PRE, [21] and have reported no deleterious effects. However, the intervention has been delivered as a home exercise programme, bringing into question the issue of compliance.

To date, a lot of studies considering exercise interventions for people with an EDSS of < 6 have a moderate to high risk of bias – mostly due to lack of blinding or not having a control group, questioning the validity of the results. More rigorous methodologies are needed to eliminate this bias and allow firm conclusions to be drawn.

Yoga is frequently provided to its members by MS Society of Ireland (MSI) and has been show to be feasible in this population[22] however, only one small scale study has evaluated it.

It is possible that the exercise needs of this group of PwMS can be met by fitness instructors in gyms providing a non medical environment for exercise participation. This study therefore aimed to assess the effect of intervention delivered by fitness instructors, following assessment by a physiotherapist to ensure that no impairments existed that would prevent this.

A review of the literature for those with an EDSS score > 6 suggests that a multitude of interventions can be beneficial in this population. These include physiotherapy, aerobic exercise and strength exercise. It is unclear, however, to what extent the results can be applied to PwMS with an EDSS score of >6 as PwMS with EDSS scores of between 1 and 6.5 were given the same interventions even though their treatment needs vary greatly. The positive outcomes were specific to the intervention used, for example, balance rehabilitation showed improvements in balance scores [12] and aerobic exercise showed improvements in fitness levels[23]. The optimal type of intervention and its frequency and duration for this population is, however, still unknown and further research is needed to develop treatment recommendations for clinicians treating PwMS with more severe mobility problems. In completing a large multi centre trial it may be possible to examine the subgroups of individuals with specific needs to establish what intervention is best for which sub groups of people with differing impairments in order to allow clinicians to deliver the optimal intervention to match the differing impairments.

The Getting the Balance Right (GTBR) projects is a collaboration between the Physiotherapy Department of the University of Limerick, and MSI that aims to both deliver and evaluate physiotherapy and exercise interventions for people with MS. There are many unknown variables to be examined in this population, therefore in order to determine the research questions with the highest priority a group of physiotherapists specialising in MS, the regional offices of MSI and the latest findings from the literature were consulted. The conclusions of this consultancy period was that the overarching question was what exercise interventions should MSI provide for their members in order to optimise function, reduce fatigue and increase quality of life.

## Methods and design

### Design

This will be a multi-centre, prospective, single blinded, block randomised controlled trial. Participants will be stratified according to mobility level using the mobility section of the Guys Neurological Disability Scale[24]. Those who score 0, 1 or 2 are stratified to stand A, those scoring 3 and 4 to strand B. For each strand blocks of subjects will be randomised to one of the interventions or to the control group.

Strand A will concern individuals with MS who walk independently or use one stick to walk outside. The objective is to compare the immediate and long term effect of yoga, physiotherapy led exercise class, fitness instructor led exercise class to a control group who don't change their exercise habits.

Strand B will concern individuals with MS who walk with bilateral support or a rollator, they may use a wheelchair for longer distance outdoors. The objective is to compare the immediate and long term effect of 1:1 Physiotherapist led intervention, group intervention led by Physiotherapist, or group yoga intervention to a control group who don't change their exercise habits.

The study protocol was approved by 10 regional ethics committees in the Republic of Ireland

### Participants

Anyone with a diagnosis of MS confirmed by their consultant physician or neurologist, resident in the republic of Ireland is eligible to take part. It is not necessary for participants to be members of MSI.

People with MS will be excluded from the study if they are currently experiencing an exacerbation of symptoms due to relapse, have had steroid treatment within 3 months of the baseline measurement, are pregnant at the time of referral, or are under 18 years of age

### Recruitment and Randomisation

Participants can be referred to the project by themselves, their carer, MSI, Chartered Physiotherapists, Neurologists, GPs or Clinical nurse specialists.

Referral will be made to the ten regional offices of MSI. A form completed over the telephone will be used to screen for exclusion criteria and to stratify according to mobility level. Participants will then be sent the information leaflet for the relevant strand and a consent form. The consent form will be returned to the regional office and consenting subjects will be coded by region, number and then strand (e.g. MW1A, MW2A, MW3B...). The participants General Practitioner will be sent a letter informing them of their participation and enclosing the relevant information leaflet. Once consent for 8 people of a strand is obtained, the regional office will contact the National Coordinator who will allocate that block of subjects to the control or an intervention condition. Block randomisation was done using a sealed enveloped with pieces of paper with the four groups written on them. The paper chosen will be emitted each time until four groups are chosen to ensure even numbers of groups. When the fifth group is ready to go, all the pieces of paper will be returned to the envelope, and so on. The order of allocation to control or intervention will be concealed from the regional offices until they have scheduled that block of 8 people for assessment.

### Assessment

Each participant will be assessed by a blinded assessor who is unaware of group allocation. Assessors will attend training days in order to standardise the measurements.

Assessments will be carried out at baseline (week 1), post-intervention (week 12) and at follow-up (week 24). The following outcome measures will be used for all three assessments: Multiple Sclerosis Impact Scale 29 version 2 (MSIS), Modified Fatigue Impact Scale (MFIS), six minute walk test (6MWT), physiological cost index (PCI), and hand held dynamometry (HHD). In addition the Berg Balance Scale (BBS) and the Modified Ashworth Scale (MAS) will be used for strand B. As equipment for PCI and HHD are not available to all assessors some participants will not have these measures recorded. The more physically demanding outcome measures were separated to avoid fatigue and so the order of testing for strand A was 6MWT including PCI, MSIS, HHD, MFIS. For strand B it was 6MWT including PCI, MSIS, BBS, MFIS, MAS, HHD

A standardised assessment at baseline considered the participants suitability for exercise and measured confounding variables such as level of sensory loss, coordination deficits etc.

#### The 6 Minute Walk Test including Physiological Cost Index

In the 6MWT, the participants will be asked to walk for a period of six minutes and the distance walked is recorded. Paltamaa et al[25] found that the 6MWT is highly reliable in people with mild to moderate MS (EDSS 2–6.5) and Marrie and Goldman[26] validated the 6MWT as an outcome measure for PwMS. Subjects will be instructed to walk as quickly and safely as possible as recommended by Fry and Pfalzer[27].

During the 6MWT the heart rate of each participant will be recorded in order to calculate the PCI. The PCI is a measure of energy is measured by calculating the heart rate during locomotion minus the resting hear rate divided by the speed of walking[28].

The exercising heart rate will be measured by a polar monitor (a strap around the chest and a watch). The resting heart rate will be measured by the patient first thing in the morning using the carotid pulse in the neck.

#### The Multiple Sclerosis Impact Scale 29 (MSIS-29)

The MSIS – 29 is a measure of the physical and psychological impact of MS from the patient's perspective. The MSIS – 29 was found to have good psychometric properties in an Irish Community dwelling population of PwMS[29]. The MSIS – 29 is an easy instrument to administer, taking approximately 5 minutes to complete. All estimates of reliability using Cronbach's alpha were in excess of the recommended 0.80. The convergent and divergent validity is established and it has moderate sensitivity to change. It has been shown to have better responsiveness compared to SF – 36 and the FAMs which also consider quality of life[30]. High scores on the MSIS-29 indicate greater impact of MS.

#### The Berg Balance Scale (BBS)

The BBS is a clinical scale that evaluates balance in sitting and standing and rates performance from 0 (cannot perform) to 4 (normal performance). The scale has fifteen items that explore the ability to sit, stand, lean, turn and maintain the upright position on one leg. The BBS has been validated for use in people with multiple Sclerosis[31]. It was found to have good concurrent validity and a cut off score of 44 (out of 56) was established as a criterion to identify PwMS who have a high risk of falls. The reliability of the BBS has also been examined in PwMS[32,33]. It was found to have high test retest and interrater reliability, both having intraclass correlation coefficients (ICCs) of 0.96. The BBS is widely used by physiotherapists and takes approximately 15 minutes to complete. It is also used in other studies evaluating interventions in PwMS[34], therefore, making it possible to compare results to other studies.

#### The Modified Fatigue Impact Scale (MFIS)

The MFIS is a structured self report questionnaire. The subscales are how fatigue relates to physical, cognitive and psychosocial aspects functioning. This version has a Cronbachs alpha of .81 indicating good reliability[35]. A recent review of the literature[36] suggested that the MFIS may have greater sensitivity to change than the Fatigue Severity Score. Administration time is approximately 5 to 10 minutes.

#### The Modified Ashworth Scale

The Modified Ashworth Scale (MAS) is a 6 point ordinal scale that is used to measure spasticity. It is designed to grade the level of resistance encountered during passive movement and scores range from 0 (no increase in muscle tone) to 5 (rigidity of the affected limb). It is widely used in clinical practice and has been shown to have good to excellent interrater and intrarater reliability in people with spasticity post stroke[37]. Its validity has been determined with the use of electromyographic recordings of muscle activity in patients with spinal cord injury [38-40]. It has also been used in other studies evaluating interventions for PwMS[41], making comparisons between results possible.

#### Handheld Dynamometry

Handheld dynamometry has been found to be more reliable than manual muscle testing (REF). The standardised positions described by Bohannon[42,43] will be used to measure strength in both the upper limbs and the lower limbs.

### Sample size calculations

#### Strand A

Based on a pilot study and the MSIS physical component scores then a sample size of 60 in each group will be sufficient to show a similar probability (0.329) that an observation after intervention will be less than before intervention as in the pilot study (mean before intervention 62.4, after intervention 53.6). This is based on using the Wilcoxon Signed Rank Test, significance level 0.05 and a power of 90%.

#### Strand B

Based on a pilot study and the MSIS physical and psychological component scores then a sample size of 22 in each group will be sufficient to show a similar probability (0.217) that an observation after intervention will be less than before intervention as in the pilot study (mean before intervention 23.0, after intervention 18.8). This is based on using the Wilcoxon Signed Rank Test, significance level 0.05 and a power of 90%.

As parametric tests require less subjects for the same power these numbers should be sufficient even if these tests are used instead of the non-parametric tests given above.

### Interventions

The physiotherapy interventions for strands A and B are standardised and based on the latest evidence for practice in order to allow reproduction of the programmes. The Yoga and Fitness Instructor interventions are not specified, in order to represent normal practice, but are well documented to allow comparison later.

To ensure standardisation of physiotherapy interventions, training for those physiotherapists delivering the programmes will be held on three occasions, supporting documentation will be provided and follow up advice and information for deliverers was supplied as necessary by e-mail and telephone. The content of the interventions for both strands is described below. The attendance and participation of each study participant will be documented on a weekly basis.

The control groups in both strands will not receive any intervention for the 12 weeks and were told to continue with their normal routine. Once the control period is over participants will receive the treatment of their choice but the response to this will not be assessed as part of this trial.

#### Strand A

##### Intervention 1 – Physiotherapy led strength and aerobic training

Participants will attend an hour long circuit class once a week for ten weeks. This will be a circuit style class consisting of Sit to stand/squat, bridging, resisted shoulder flexion, walking/bike, resisted elbow flexion, lunges or resisted knee extension, hip extension, calf raises. All exercises will be completed at 50 – 80% of the participants' one-repetition-maximum and the load will be increased by 2 – 5% when twelve repetitions are easily achieved. The aim is for the participant to be failing on the last repetition. If the next available load is higher than 2 – 5% (such as going from 1 kg to 2 kg in resisted shoulder flexion) the participant can increase the repetitions so that they are still failing to achieve full range on motion on the last one. The primary aim is to achieve "overload" of the muscle to achieve gains in strength. Participants will be educated about the overload principle and that delayed onset of muscle soreness is a normal phenomenon. After five weeks of attending the class, participants will be advised to complete a second hour of strengthening themselves at home.

A second component of the class will be advice regarding aerobic exercise. In the first class, 10 minutes will be devoted to formally explaining aerobic exercise, Uhtoff's phenomenon (whereby increasing heat in people with MS can temporarily exacerbate symptoms) and the evidence supporting aerobic activity.

Each participant will be given a target heart rate. This will be calculated using the Karvonen formula which is: Target Heart Rate = Resting Heart Rate + 65% (Maximum Heart Rate).

The participants will be advised in the initial assessment how to get their resting heart rate, by feeling their carotid pulse for 30 seconds first thing on waking. The first beat they feel will be zero. As a maximal exercise test will not be conducted, each participant's age predicted maximum heart rate will be used as the target HR (i.e. 220 – age). If they are unable to detect a pulse they will aim to exercise at a Rate of Perceived Exertion level of 11 – 14.

Participants will be initially asked to complete two sessions of independent aerobic activity per week for twenty – thirty minutes. From week 5, they will be asked complete this three times a week. Participants can choose the type activity that they did (i.e. swimming/walking/running/cycling). They will take their pulse every ten minutes to ensure they are achieving the correct intensity.

##### Intervention 2 – Fitness Instructor led group exercise

The fitness instructors be registered by the National Council for Exercise and Fitness. They will be given a standardised pack in order to document the interventions.

##### Intervention 3 – Yoga classes

Yoga instructors will be accredited by the Yoga Federation of Ireland. They will be given a standardised pack in order to document the interventions.

#### Strand B

##### Intervention 1 – Group Physiotherapy

Participants attended a weekly hour long class for 10 weeks. The circuit style class will consist of exercises that combine strength and balance. Participants will complete 6 different exercises at each class. The exercises were adapted from the falls prevention literature where similar programmes have been seen to improve, balance and reduce the number of falls in an elderly population.

The six exercises and possible progressions are described below. These are to be performed in sets of 12. When a participant is able to perform 12 repetitions of an exercise safely this can be progressed up to 3 sets of 12 repetitions. When a participant can perform 3 sets safely the exercise will be progressed so as to continuously challenge the participant. Not all participants will progress through all the exercises. The progression is dependent on the ability of the participant and their safety while performing the exercises

1. Sit to Stand, progressed by altering;

Hand Positioning – Participants may initially need to use hands for support to rise from chair, then progressing to hands by side and then to hands across chest.

Seat Height – Participants may initially require a higher seat height which can be lowered to increase the intensity of the exercise.

Repetitions – To be performed in sets of 12 and number of sets to be increased to 3 as participant progresses.

Weights – Handheld weights may be given to participants who need further progression.

2. Squat, progressed by altering;

Support – Participants may initially need bilateral support, this can be decreased to unilateral and then to no support as participants' ability increases.

Repetitions – To be performed in sets of 12 and number of sets to be increased to 3 as participant progresses.

Weights – May be given to participants who are able to perform 3 sets of 12 squats safely with no support.

3. Calf Raises, progressed by altering;

Support – Participants may initially need bilateral support, this can be decreased to unilateral support and then to independent calf raises as participant progresses.

Repetitions – To be performed in sets of 12, to be increased as participant progresses.

Other options – If participants are able they may perform single leg calf raises or if they can perform 3 sets of 12 independent calf raises weights can be added as further progression.

The following three exercises are to be completed within parallel bars.

4. Step ups, progressed by altering;

Support – Participants may begin with bilateral support, and then decrease to unilateral support, then to no support.

Stepping – Initially participants may step onto step and back to starting position, then step onto step and over, and then onto step, over and backwards to starting position.

Step Height – When participants are comfortable with all directions of stepping step height may be increased.

5. Side Stepping – progressed by altering;

Support – Participants may begin with bilateral support, and then decrease to unilateral support, then to no support.

Number of steps – Initially participants may only take one step in each direction. This can be increased as participants' ability increases.

If a participant is unable to take a step to the side, weight shifting from side to side in standing may be performed and progressed to stepping when the participant is able.

6. Tandem Stepping/Walking – progressed by altering;

Support – Participants may begin bilateral support, and then decrease to unilateral support, then to no support.

Stepping – Participants may initially just place one foot in front of the other and hold this position. The number of steps can then be increased as the participant progresses.

Crossover – Participants may become competent at tandem walking. This can then be progressed to one foot crossing over in front of the other.

##### Intervention 2 – One on one Physiotherapy

These participants will receive individual treatment depending on the problem list and goals established by the Chartered Physiotherapist who was treating them. The content of the intervention will be recorded for each individual treatment session. The duration of the individual sessions will be the same as the group led physiotherapy.

##### Intervention 3 – Yoga

Participants will attend a weekly yoga class of approximately one hour's duration. All yoga instructors will be members of The Yoga Federation of Ireland and will keep a log of the content of each yoga class.

### Statistical Analyses

All measures will be summarised and tested for normality. If variables are non-normal then Wilcoxon Signed Rank tests will be used to test for differences before and after interventions and Mann-Whitney U tests will be used to test for differences between groups before and after interventions. If the variables are reasonably normally distributed then repeated measures ANOVA will be carried out to test for differences between groups and over time. Suitable plots will be used to demonstrate the changes found.

## Discussion

This multi-centre, prospective, single blinded, block randomised controlled trial aims to determine which physiotherapy or exercise intervention MS Ireland should deliver in order to reduce the impact of MS, reduce fatigue and optimise activity in people with MS. It uses the recommendations of the Cochrane review[6] by including blinded assessments, concealing allocation to groups and documenting drop outs in detail. If sufficient numbers are recruited sub-group analysis may be carried out in order to analyse which impairments are associated with success from each of the interventions, or who does best from what treatment.

## Competing interests

The authors declare that they have no competing interests.

## Authors' contributions

SC is PI for the trial and wrote this paper with contributions from all authors. MG and NH are PhD students working on strand A and B respectively. They conducted the literature reviews and developed the study protocol under the supervision of SC. AL is project coordinator for MS Ireland and contributed to the practical aspects of study design and implementation and proof read this paper. JS is Executive Director of the Statistical Consulting Unit and performed power calculations and gave advice on statistical analysis of the data.

## Pre-publication history

The pre-publication history for this paper can be accessed here:


